# Scaling and biomechanics of surface attachment in climbing animals

**DOI:** 10.1098/rstb.2014.0027

**Published:** 2015-02-05

**Authors:** David Labonte, Walter Federle

**Affiliations:** Department of Zoology, University of Cambridge, Cambridge, UK

**Keywords:** bioinspiration, contact splitting, controllable adhesion, wet adhesives

## Abstract

Attachment devices are essential adaptations for climbing animals and valuable models for synthetic adhesives. A major unresolved question for both natural and bioinspired attachment systems is how attachment performance depends on size. Here, we discuss how contact geometry and mode of detachment influence the scaling of attachment forces for claws and adhesive pads, and how allometric data on biological systems can yield insights into their mechanism of attachment. Larger animals are expected to attach less well to surfaces, due to their smaller surface-to-volume ratio, and because it becomes increasingly difficult to distribute load uniformly across large contact areas. In order to compensate for this decrease of weight-specific adhesion, large animals could evolve overproportionally large pads, or adaptations that increase attachment efficiency (adhesion or friction per unit contact area). Available data suggest that attachment pad area scales close to isometry within clades, but pad efficiency in some animals increases with size so that attachment performance is approximately size-independent. The mechanisms underlying this biologically important variation in pad efficiency are still unclear. We suggest that switching between stress concentration (easy detachment) and uniform load distribution (strong attachment) via shear forces is one of the key mechanisms enabling the dynamic control of adhesion during locomotion.

## 1. Introduction

**Figure 1.**
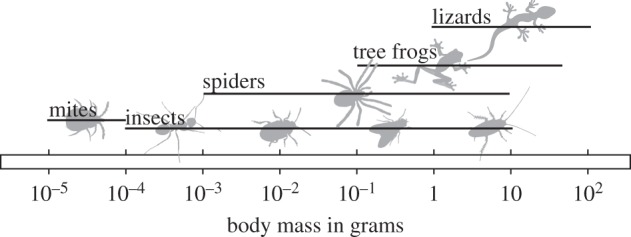
Animals that use adhesive pads for climbing span approximately seven orders of magnitude in body mass.

The ability to climb on plants and in the canopy of trees conveys significant ecological advantages and is widespread in the animal kingdom. The largest climbing animals can move in trees by grasping around stems and branches with their long limbs and hands [1]. For smaller vertebrates and arthropods, however, thicker stems and branches are effectively ‘flat’ surfaces, and climbing requires specific attachment structures such as claws and adhesive pads, which have evolved convergently in many groups of arthropods, lizards and tree frogs [2–11]. The body mass of animals climbing with adhesive pads varies over about seven orders of magnitude from the smallest mites to the largest geckos (figure 1), and larger animals face two problems related to their size: (i) As the ‘foot’ area available for attachment structures scales with an animal's surface area, it grows more slowly than body mass. (ii) For larger animals, it may be more difficult to avoid stress concentrations and thereby distribute stresses uniformly across the contact zone, so that forces may not be proportional to contact area (and thus *m*^2/3^ for isometric animals, where *m* is body mass), but to even lower powers of mass. As a result of both factors, larger animals are expected to have smaller ‘safety factors’ (maximal sustainable force per body weight), making it more difficult for them to support their body weight when climbing. It is likely that the design of biological attachment structures has been adjusted in the course of evolution to compensate for this expected loss of weight-specific adhesion, using one or a combination of two strategies: (i) larger animals could develop overproportionally large pads or (ii) their pads could become more efficient, i.e. they could sustain a larger force per area.

Clarifying how larger climbing animals respond to the hypothesized loss of weight-specific adhesion may also provide insights relevant for the development of bioinspired synthetic adhesives, which has recently attracted considerable attention (for reviews, see [12–14]). Many applications for such synthetic adhesives require controllable adhesion over areas larger than those of natural adhesive systems. Therefore, one of the key challenges in the fabrication of bioinspired adhesives is the up-scaling from micrometre-sized structures to macroscopic systems. In fact, most existing mimics have failed to show sufficient adhesion at macroscopic length scales [15,16]. Analysing attachment structures in organisms of different size is thus important to reveal potential solutions.

In this study, we investigate the size dependence of biological attachment by comparing predictions for different attachment mechanisms with existing information on the scaling of animal attachment devices and their performance. Most attachment mechanisms are size-dependent, but the expected scaling coefficients depend on the contact geometry and the mode of detachment. As the mechanisms of animal adhesion are far from being fully understood, studying the allometry of attachment structures and the scaling of attachment performance may help to discriminate between different hypotheses.

## 2. Scaling of claws

Claws are probably the most widespread attachment structures and can be found on the feet of most climbing mammals, birds, lizards and arthropods, suggesting that they are excellent clinging tools. Most previous work on animal claws and climbing was conducted on birds and lizards and focused on the relationship between claw shape and habitat or ‘lifestyle’ [17–21]. For example, climbing birds were found to have more strongly curved claws than ground-dwelling ones [19], claws of arboreal anoles are longer and have a larger ‘base height’ than those of non-arboreal anoles [21], and claw curvature correlated with clinging force in lizards [18], suggesting a functional advantage. Zani [18], Tulli *et al*. [22] and Crandell *et al*. [21] reported a positive correlation between ‘claw base height’ and clinging force, but the underlying mechanism was not discussed. In general, the relationship between claw morphology, substrate characteristics and clinging performance remains poorly understood.

Claws are made of stiff and hard cuticle or keratin [23–25] and are probably more wear-resistant than adhesive pads [26], which have to be compliant in order to make contact to rough surfaces [27,28]. As a result of their high stiffness, however, the friction coefficient *μ* of claws on rigid, smooth surfaces may be relatively small (for sclerotized cuticle on glass *µ* ≈ 0.35, see [23]), so that claws only represent an advantage over adhesive pads if they are either able to interlock with surface asperities, or if they significantly indent and/or penetrate the substrate.

Dai *et al*. [23] modelled the grip of claws on rough surfaces as the interaction between a conical claw with a hemispherical tip and a rigid hemispherical surface asperity. Although the geometry is simplified, the model qualitatively captures the performance of claws on rough surfaces [23,29]. The key factor determining the claw's ability to interlock is the sharpness of its tip, which may be defined by the radius of the claw tip *R*_CT_. If the hemispherical asperities are much larger than this radius, claws can interlock and withstand large forces, limited by the mechanical strength of the claw material. The design of claw tips may thus be the result of a trade-off: claws should be sharp to maximize the probability of finding surface asperities that are small enough to allow interlocking, but should be sufficiently thick (and therefore blunt) in order to minimize the risk of fracture and wear [30]. For rough substrates such as stone or concrete, the number of ‘usable’ asperities per unit length varies approximately with 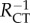 [30]. The maximum stress before failure, in turn, increases with 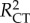, independent of whether the claw tip or the asperity fails [30].

Assuming isometric scaling of the claw tip radius, larger animals would therefore face two significant problems when engaging claws during climbing: their claws would not only find fewer ‘usable’ asperities, but they would also fracture and wear more easily due to the smaller ratio of claw tip radius to body weight. This problem is potentially aggravated by the fact that the number of usable asperities decreases with the claw tip radius only up to a surface-specific cut-off length, above which it decays even more quickly. Stones, for example, have only few larger asperities, as they are smoothed by wind and water [30]. We are not aware of any study of claw tip diameter in relation to body weight, and thus it remains unclear how climbing animals cope with this problem, and whether claws of larger animals are indeed worse at gripping on substrates with small asperities. If selection favours a constant number of usable asperities per unit length, claw tip radii should be negatively allometric. If avoiding claw tip failure is the key constraint for claws, however, one would expect their design to maintain a constant stress at the tip, in which case the tip radius would show positive allometry and scale with *m*^0.5^ (see appendix A).

Independent of body weight, the wear or failure of claw tips may have substantial consequences for animals relying heavily on their claws during climbing [31–34]. Two potential adaptations may help to reduce claw damage and wear: first, in order to distribute load equally among multiple engaged claws, they should be flexibly hinged at their base [30,35], as is indeed the case for insects and lizards [36–38]. Second, animals may incorporate particularly wear- and fracture-resistant materials at the tips of their claws. Many arthropods show high concentrations of metals in structures that are exposed to abrasion and high mechanical stress. Metal inclusions have been reported for the mandibles/mandibular teeth in several insect orders and spiders [39–45], the stings of scorpions [44], the ovipositors of some hymenopteran insects [46,47] and indeed the claws of some insects and scorpions [42,44]. Inclusion of zinc has been shown to increase the hardness and stiffness of cuticle [39,43,45], but it remains unclear whether the presence and concentration of metals vary with body weight.

## 3. Scaling of adhesion

Adhesion refers to the attachment of different materials at their interface. In this review, we refer to ‘adhesive forces’ as separation-resisting forces that act *normal* to the interface and to ‘friction’ as forces resisting movement *parallel* to the interface. If detachment occurs by the propagation of an interfacial ‘crack’ driven by stress concentrations near the crack tip, adhesion may be measured as the work required to detach a unit area of the interface, commonly referred to as the *effective work of adhesion*, which can be much larger than the thermodynamic work of adhesion. If detachment stresses are distributed uniformly across the interface, adhesion may be quantified as a *contact strength*, i.e. the force required to separate one unit area. In this paper, we will use both measures, depending on the context.

The adhesion of attachment pads depends on the type of intermolecular forces involved, the geometry of the adhesive contact and the mode of detachment. Independent of contact geometry, the pull-off force is limited by the theoretical strength of the adhesive interaction. Previous research has demonstrated that dynamic, biological adhesives use ‘weak’ bonds via long-range van der Waals forces, or via the surface tension of a fluid [48–54]. For van der Waals forces, the theoretical contact strength can be as high as 20–200 MPa [55,56]. Usually, the actual pull-off forces are much smaller than predicted from the theoretical contact strength, as small ‘defects’ weaken the interface locally, and stresses during detachment are concentrated in a narrow peel zone at the periphery of the contact. In such cases, adhesion will not scale with contact area (and thus with *m*^2/3^ for isometric animals), but with lower powers, and will be sensitive to the geometry of the contact.

Despite the diversity of climbing animals, their adhesive structures come in only two basic designs. They are either soft pads with a macroscopically smooth surface profile, or ‘hairy’, i.e. densely covered with micrometre- or nanometre-sized setae. Some pads are effectively fluid-filled membranes, while others are more compact structures [57]. The shapes of individual hairs in fibrillar pads show a similar diversity, including non-branched and branched setae, terminated by mushroom-shaped, spatula-like or pointed/conical tips [6,51,58].

Previous authors have derived predictions for pull-off forces for different contact geometries, including flat, spherical, conical, toroidal and ‘mushroom-shaped’ tips [55,56,59,60], liquid-filled membranes [61] and thin blades which peel like Scotch tape [62,63], as well as for the influence of capillarity and viscosity in ‘wet’ adhesive contacts [64–67]. Each of these models predicts a specific dependence on the dimensions of the contact, and thus the scaling of attachment forces depends both on the contact geometry and mode of detachment.

### (a) Length scaling

**Figure 2.**
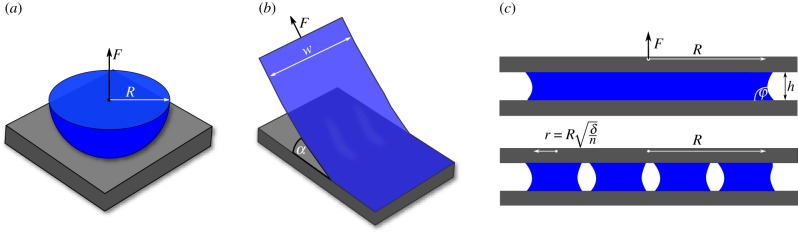
Schematic drawings of loading geometries that are frequently used to model biological attachment pads. (*a*) An elastic sphere of radius *R* in contact with a surface. This case is considered with or without the presence of a contact-mediating liquid. (*b*) A thin tape of width *w* is peeled off a substrate at an angle *α*. (*c*) Two rigid plates separated by a thin film of liquid of radius *R*. Instead of a single meniscus, the available area can also be filled with multiple smaller menisci with radii 
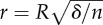
, where *n* is the number of menisci, and *δ* is their area coverage.

Length scaling can occur when the separation process is confined to a region smaller than the contact area. Several authors have modelled biological adhesive pads as spherical contacts or thin tapes [53,56,68–73] (figure 2*a*,*b*). Contact mechanics models for soft and rigid spheres, for spheres in the presence of a liquid, and for liquid-filled spherical membranes predict pull-off forces linearly proportional to the sphere's radius of curvature [59,61,66,74,75]. Length scaling is also predicted for the steady-state peeling of thin films, where forces are proportional to the width of the peeled strip of elastic tape [62,63].

### (b) Area scaling

Area scaling will occur if stresses are uniformly distributed across the contact zone. A classic example for area scaling of adhesion are suction cups, where a low pressure is produced underneath the cup. A low pressure within the adhesive contact zone can also be produced by the surface tension of a liquid. On wettable surfaces (i.e. contact angles less than 90°), the meniscus at the contact perimeter will be concave, resulting in a pressure difference across the interface (lower inside the fluid) according to Laplace's law. For a fluid film between two rigid discs, this low pressure will again be uniformly distributed across the contact (as for rigid materials, the surface tension of the liquid is not sufficient to deform the plates), and pull-off forces are predicted to scale with the area of contact (assuming a size-invariant fluid film thickness, see figure 2*c* and appendix B).

Uniform load distribution can also be achieved if a single contact is smaller than a material- and geometry-specific critical crack length [55]. Even for contacts larger than the critical crack length, a uniform load distribution can be achieved if the contacts are slightly concave, but minor departures from this ideal geometry can result in a large decrease in pull-off force [55]. Some of the most successful examples of bioinspired technical adhesives are ‘mushroom-shaped’ adhesives, inspired by the adhesive hair tips found in some beetles [76–80]. Owing to their specific geometry, these tips are less sensitive to defects close to the contact periphery (edge), so that cracks initiate in the centre of the contact, and stresses are more uniformly distributed [60,81,82].

### (c) Between length and area: intermediate scaling

For some contact geometries such as a torus or a flat punch, pull-off force shows an intermediate scaling between length (*m*^1/3^) and area (*m*^2/3^ for isometric animals) [56]. Assuming that adhesion can be modelled as an exchange between surface energy and elastic energy, Bartlett *et al*. [16] predicted that adhesive force scales as3.1
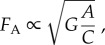
where *G* is the effective work of adhesion, *A* is contact area and *C* the compliance of the adhesive in the loading direction, and good agreement with measurements on synthetic adhesives and some data on biological adhesives was found. Similar expressions can be obtained from Kendall's peel model [63] for 0° peeling (i.e. pure shear-failure) or Griffith's criterion for flat-ended fibres [83]. Assuming that the adhesive is a flat punch of cross-sectional area *A* and length *L* along the pulling direction, made of a homogeneous material of elastic modulus *E*, the compliance would be *C* = *L*/(*EA*) and adhesive force as defined by equation (3.1) would scale as3.2
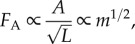
for isometric animals, i.e. intermediate between length and area scaling. The same scaling would be obtained for other geometries of shear loading [84,85].

### (d) Above-area scaling

One situation where adhesion grows faster than contact area is the separation of two rigid, parallel discs immersed in a Newtonian fluid [64]. Detachment (disc separation) requires a flow of fluid into the increasing gap, and the resulting viscous forces depend on the detachment speed and the thickness of the fluid film. Assuming that the fluid's viscosity and the film thickness are independent of pad (disc) size, detachment forces are predicted to scale with the square of contact area, or with *m*^4/3^ ([64]; see the electronic supplementary material). If film thickness is proportional to contact diameter, however, adhesion should scale with length (see §5, table 1 and appendix B).

**Table 1.** Scaling predictions for ‘wet’ adhesion models. Scaling coefficients are given for the assumption that fluid film thickness *h* depends on contact radius *R* as *h* ∝ *R^H^*, with 0 ≤ *H* ≤ 1 (see appendix B and the electronic supplementary material for details).

force type (geometry)	scaling coefficient
Laplace pressure (rigid plates)	*m*^(2−*H*)/3^
viscous (Stefan) adhesion (rigid plates)	*m*^(4−3*H*)/3^
viscous adhesion (sphere)	*m*^(2−*H*)/3^
friction (rigid plates)	*m*^(2−*H*)/3^

### (e) Increasing pad efficiency by contact splitting

Several authors have modelled the tips of adhesive hairs as thin tapes or spherical contacts [53,56,68,86], implying length scaling of adhesion. It has been proposed that if the adhesive force of a single fibril scales with length, the force per area for a fibrillar array should increase for smaller sized contacts since *F*_A_/*A* ∝ *L*/*L*^2^ = 1/*L* [53], and that larger animals could thus have developed denser arrays of smaller adhesive hairs in order to compensate for the weight-specific loss of adhesion [68,86]. In the following, we will refer to this prediction as ‘force scaling’ to distinguish it from other benefits associated with a decrease of the size of individual contacts (‘contact splitting’, e.g. [15,87,88]). The idea that fibrillar adhesives can benefit from decreasing the size of individual contacts is appealing and has attracted much attention [15,55,56,68,87–94].

When one large contact with radius *R* is divided into *n* sub-contacts, each of radius *r*, the total area covered by the sub-contacts is inevitably smaller than the available contact area. Thus, the force *F*_C_ after contact splitting is [13,91]3.3
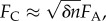
where *F*_A_ is the adhesive force prior to contact splitting and *δ* = *nr*^2^/*R*^2^ is the area fraction covered by the contact elements. For fibrillar (unbranched) adhesives, the upper limit of *δ* is around 0.3–0.5, as seta density is limited by self-matting [69,95,96].

Equation (3.3) implicitly assumes stress concentrations and length scaling at the level of each individual contact, but uniform load distribution across the whole adhesive pad, so that all contact elements are pulled off simultaneously and the pad's adhesive force is equal to the force of a single seta multiplied by the number of setae. If pull-off forces of individual contacts scaled with area, however, splitting a large contact into many smaller ones would lead to a *decrease* in adhesive force, due to the assoiated loss of total contact area. For above-area viscous scaling, contact splitting would reduce adhesion even if total contact area remained constant.

If length scaling occurs not only for individual contacts but also at the level of the whole pad (i.e. the pad detaches by peeling), contact splitting could still increase pull-off forces if it increases the array's effective work of adhesion (i.e. the energy per unit pad area required for detachment). As adhesive hairs act as ‘crack arresters’ [89,97], the effective work of adhesion of a setal array corresponds approximately to the energy per area required to detach a single seta. This energy increases for denser arrays of longer, thinner and softer setae, but such morphological changes are limited by the increasing tendency of setae to stick to one another (self-matting). This self-matting constraint is expected to nullify any gain in effective work of adhesion by contact splitting, unless a branched morphology is developed as in spiders and geckos, which can prevent self-matting even for very fine setae [87].

An upper limit of ‘force scaling’ is set by the critical crack length, which in turn will depend on the adhesive's shape, stiffness and effective work of adhesion (e.g. [55]). If the size of individual contacts is comparable to this critical length, the adhesive approaches the theoretical strength of the respective intermolecular interaction [55]. In terms of scaling, decreasing the size of individual contacts thus corresponds to a transition from length scaling (stress concentration) to area scaling (uniform stress) [13,69]. As a consequence, ‘force scaling’ can only increase adhesion until individual contacts are in the area scaling regime [13,15].

A different constraint for increasing strength via contact splitting occurs for ‘wet’ contacts, where the adhesive force can be the sum of an area-specific (Laplace pressure) and a length-specific (surface tension) term (see figure 2*c* and appendix C). Previous studies suggested that decreasing individual contact size may help to increase the force per area for such ‘wet’ adhesives [92,93,98]). Assuming rigid and flat contacts, and a size-invariant fluid film thickness, however, adhesion gain by contact splitting is only possible for contact angles larger than 30°, provided that the number of sub-contacts *n* is sufficient to balance the loss of adhesion due to the smaller total contact area (see appendix C, and figure 7*a*). As the curved fluid menisci require a minimum diameter for a given fluid height, *n* is limited in the case of size-invariant fluid film thickness, setting an even more restrictive condition than *ϕ* > 30° for adhesion gains via contact splitting (see appendix C, and figure 7*b*).

The pad secretions of insects, spiders and tree frogs wet both hydrophilic and hydrophobic surfaces, with contact angles smaller than 30° [99–101]. This would suggest that biological fibrillar adhesives are outside the range where ‘wet’ contact splitting can increase adhesion. However, assuming that film thickness is proportional to hair diameter (see appendix C), both Laplace pressure and surface tension would scale with length, and contact splitting would result in adhesion gains for all contact angles less than 90° (appendix C).

## 4. Scaling of friction

As stiff solids are typically rough at least on a microscopic scale, only their highest asperities can come into close contact. As a consequence, contact area is essentially zero in the absence of load and increases approximately linearly with normal force, resulting in a linear load dependence of friction [102–104]. Load-dependent friction (Coloumb friction) is therefore expected to scale with mass.

Adhesive pads of climbing animals are very soft [27,28,105], so that intermolecular forces are sufficient to produce a significant contact area even for small or negative loads [106–108]. As a result, the pads' resistance against sliding is largely independent of normal load which is essential for climbing animals. In this ‘adhesion-controlled’ regime of friction, forces are expected to scale with area. Area scaling of friction is also predicted for the contribution of viscosity in ‘wet’ contacts, such as two flat, rigid plates separated by a thin liquid film [109,110]. As before, this prediction applies only if the fluid film's thickness is independent of pad size. If film thickness scales with contact diameter, friction would scale with length (see §5, table 1 and appendix B).

The shear forces of ‘wet’ contacts are also influenced by the fluid's surface tension. In contrast to viscosity, surface tension provides a *static* resistance against sliding, even for purely Newtonian liquids. If two plates joined by a liquid meniscus slide relative to each other, the fluid's contact angle *ϕ*_1_ at the ‘trailing edge’ may differ from that at the ‘leading edge’*ϕ*_2_, an effect commonly referred to as contact angle hysteresis. This hysteresis leads to an asymmetric deformation of the meniscus during sliding, which results in a ‘restoring force’ *F* [109]4.1
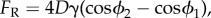
where *D* is the width of the contact zone, and *γ* is the surface tension of the liquid. As the restoring force scales with the width of the contact, it is negligible for large contacts, but can become significant for dense arrays of smaller contacts. While the terminal elements of setae can have many different shapes [51,58], spatula-tipped setae represent the most common design [51]. For spatulae, 0.2 µm < *D* < 10 µm [111] and hence equation (4.1) predicts maximum possible static shear stresses of 8 *Dγ* = 15–760 kPa (assuming maximum hysteresis and a surface tension of 30 mN m^−1^). Thus, surface-tension-based shear forces can be increased by contact splitting. While it has been observed that surface structuring can change the scaling of friction forces [112], the principle of contact splitting has so far been discussed only in the context of adhesion.

## 5. The importance of fluid film thickness for the scaling of ‘wet’ adhesion and friction

The scaling of friction and adhesion of ‘wet’ contacts depends not only on the specific contact geometry and the type of forces involved, but also on the relationship between fluid film thickness and contact size. Previous analyses have assumed that fluid volume and total contact area are conserved so that fluid film thickness is size-invariant [92,93]. However, as one of the functions of the pad secretion is to mediate adhesion on rough surfaces [106], it is likely that larger contacts require thicker fluid films and it may be assumed that film thickness is linearly proportional to contact size. A more general scaling prediction can be derived by assuming that the amount of fluid is sufficient to compensate the surface roughness amplitude of a self-affine rough surface. On smooth surfaces, insects should secrete as little fluid as possible to maximize pad adhesion, while on rough surfaces the secretion film should be thick enough to fill the gaps in order to increase adhesive forces [106,113]. This condition is satisfied if *h* ∝ *R^H^*, where 0 ≤ *H* ≤ 1 is the Hurst exponent, *R* is the radius of the circular contact and *h* is the thickness of the fluid film (see appendix B). The scaling of both friction and adhesion is predicted to depend on *H* and can vary from length to above-area (table 1).

## 6. Scaling of biological adhesive structures and their performance

### (a) Scaling of adhesion and contact area in leaf-cutting ants

**Figure 3.**
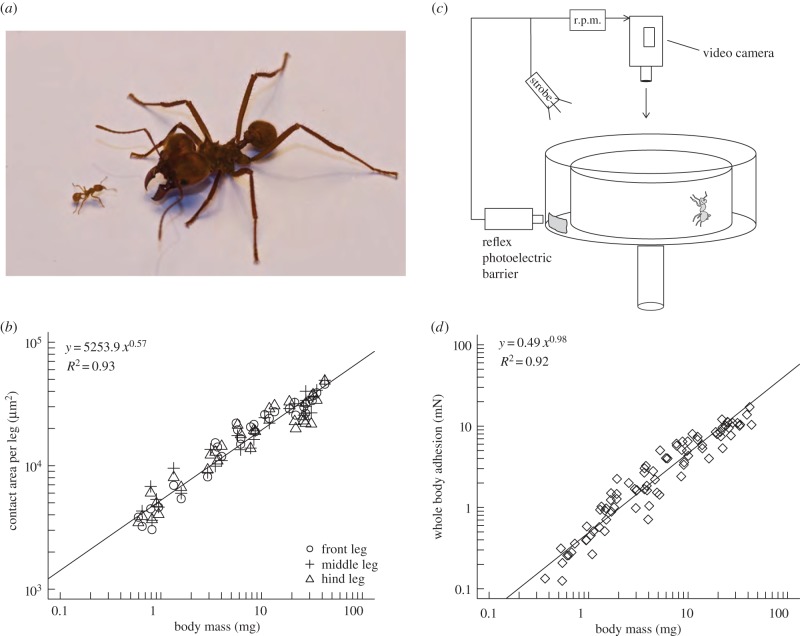
(*a*) Leaf-cutting ants (here: *Atta cephalotes*) can differ considerably in body size, covering more than two orders of magnitude in mass. (*b*) A centrifuge technique was employed to investigate how adhesion of *A. colombica* ants depends on body size. (*c*) Pad contact area was negatively allometric for hind, middle and front legs. (*d*) Despite the negative allometry of pad area, adhesive force was almost directly proportional to body mass. The straight lines in (*c*,*d*) are the result of a SMA regression on log-transformed data. (Online version in colour.)

In order to investigate how whole-animal adhesive forces depend on body size, and how the observed effects relate to the allometry of adhesive structures, we collected an extensive dataset for leaf-cutting ants (*Atta colombica*), combining measurements of both pad area and whole-body adhesion force (see figure 3 and the electronic supplementary material for details). The ants' body weight ranged over more than two orders of magnitude from 0.37 to 43.40 mg. Scaling coefficients for pad contact area obtained from standardized major axis (SMA) regression did not differ significantly between front, middle and hind legs (likelihood ratio statistic = 2.88, d.f. = 2, *p* = 0.24), and the common slope of 0.55 was significantly smaller than the value of 2/3 expected for isometry (95%; confidence interval 0.51–0.58, *r*_89_ = −0.51, *p* < 0.001; figure 3*c*).

Measurements of the ants' attachment force revealed a scaling coefficient of 0.98 (95%; confidence interval 0.92–1.04), significantly exceeding the prediction for area scaling (2/3, *r*_82_ = 0.8, *p* < 0.001; figure 3*d*). As the confidence interval of the SMA regression includes 1.0, the ants' force per body weight appears to be almost independent of body size.

The results clearly indicate that the scaling coefficients for adhesive performance and adhesive pad area differ considerably, demonstrating that adhesive strength increases with body size. Thus, leaf-cutting ants compensate for the predicted loss of weight-specific adhesion not by a positive allometry of their adhesive pads, but by an increase of pad efficiency.

### (b) Allometry of adhesive pads

A wider survey of available data on the scaling of adhesive pad area in different climbing animals is given in table 2. Consistent with the above findings for leaf-cutting ants, neither lizards, tree frogs nor other insects appear to compensate the predicted loss of adhesion by positive allometry of their adhesive pads. Most datasets for intraspecific scaling (labelled with ‡) suggest even negative allometry of adhesive pad area.

**Table 2.** Scaling of pad area, as well as adhesion and friction forces with mass (*m*)**,** body length (*L*) or pad area (*A*) for various groups of climbing animals. Note that the contact area data for hairy pads refer to ‘projected contact area’, i.e. they do not account for changes in hair-tip size or density. When scaling coefficients were not explicitly given (labelled with †), data were extracted using a web-tool (WebPlotDigitizer by Ankit Rohatgi, http://arohatgi.info/WebPlotDigitizer/), and scaling coefficients were estimated by performing reduced major axis regression on log-transformed values. Intraspecific data are labelled with a **‡**. Where available, we give a range of the regression coefficients.

	animal	scaling	source
toe pad area	lizards (fibrillar pads)	*m*^0.59^	[114]
		*m*^0.75^–*m*^0.78^	[115]
		*m*^0.60^–*m*^0.80^	[116]
		*m*^0.57^–*m*^0.73^‡	[117]
	tree frogs (smooth pads)	*m*^0.61^–*m*^0.76^‡	[118]
		*m*^0.65^–*m*^0.90^	[118]
		*L*^1.85^	[119]
		*L*^1.76^–*L*^2.29^‡	[120]
		*L*^1.88^	[121]
	insects (smooth pads)	*m*^0.27^‡	[122]
		*m*^0.62^‡	[109]
		*m*^0.58^‡	[123]
		*L*^1.68^–*L*^2.19^‡	[124]
		*m*^0.54^‡	[125]
		*m*^0.51^–*m*^0.58^‡	this study
	insects (fibrillar pads)	*m*^0.81^†	[126]
		*m*^0.42^–*m*^0.70^†‡	[127]
adhesion	tree frogs (smooth pads)	*A*^1.19^	[119]
		*L*^2.12^–*L*^3.1^‡	[120]
		*L*^2.19^	[121]
	insects (smooth pads)	*m*^0.67^–*m*^0.90^	[128]
		*m*^0.5^‡	[122]
		*m*^0.39^‡	[122]
		*m*^0.38^‡	[122]
		*m*^0.62^	[122]
		*m*^0.57^–*m*^0.87^	[124]
		*m*^0.92^–*m*^1.04^‡	this study
friction	lizards	*m*^0.9^–*m*^0.95^	[115]
		*m*^0.45^–*m*^0.65^	[116]
	tree frogs	*L*^2.7^	[119]
	insects (fibrillar pads)	*m*^0.74^†*m*^0.84^–*m*^1.56^†‡	[126] [127]
	insects (smooth pads)	*m*^0.71^‡	[123]

### (c) Scaling of adhesion

The available scaling coefficients for adhesive forces in different climbing animals vary considerably and include near mass-, area- and close to length scaling (table 2). We generally find only little evidence for length scaling of adhesive forces, suggesting that contact models predicting length scaling should not be used at the whole-animal level.

The presented data all refer to ‘whole-animal’ measurements, but they were gathered under different conditions (e.g. with different test substrates and varying pull-off speeds) and are therefore difficult to compare. In many cases, the scaling of pad efficiency cannot be directly inferred from the data in table 2, as the regressions were performed against body length or mass, and not against pad area. Nevertheless, some of the data do indicate an increase in pad efficiency with body size, i.e. that attachment forces change faster than pad area (scaling coefficients more than 2 for length, more than 1 for area or more than 0.66 for mass). In vetch aphids, mass-specific scaling coefficients for adhesion were less than 0.66, but coefficients for contact area were even smaller, again suggesting an increase in pad efficiency [122].

### (d) Scaling of friction

Available data on the scaling of ‘whole-animal’ friction forces are presented in table 2. In most studied animals, friction appears to increase faster than expected for contact area dependence (*m*^2/3^ for isometric animals), but slower than expected for load dependence (*m*^1.0^). Notably, friction forces of individual adhesive pads on smooth surfaces are largely independent of normal load [107,108,129], indicating that the pads make intimate contact over almost the whole available contact area at near-zero loads, so that additional load can only slightly increase friction. Friction of adhesive pads should thus scale with area, even when normal loads are positive. The tendency of some coefficients to exceed the expected area scaling suggests that—as for adhesion—larger animals possess yet undiscovered strategies to increase their pads' shear stress.

## 7. Discussion

The question of how attachment forces scale with the size of animals is fundamental to the functional understanding of biological attachment systems and has direct implications for the development of bioinspired adhesives. The performance of claws and adhesive pads is affected by physical constraints which may be increasingly limiting for larger climbing animals. While the scaling of claw sharpness (claw tip diameter) with body size is still unknown, some conclusions can be drawn from available data on the allometry of pad size, adhesion and friction (summarized in table 2). The results indicate that—at least within taxa—adhesive pad area tends to scale isometrically or is even negatively allometric, while adhesive force and friction scale with area or even higher powers. This finding raises two important questions: first, for most adhesive mechanisms, area scaling of adhesion can only be achieved if the area over which stresses are distributed during detachment is comparable to the size of the pads. How are animals able to reduce stress concentrations and achieve an almost uniform stress distribution? Second, our results indicate that at least in some animal taxa, adhesion grows faster than pad area, i.e. the pads' efficiency (force per unit area) increases with body size. What is the mechanism of this biologically important increase?

In the following sections, we will first examine the parameters that influence the size of the ‘peel zone’ (i.e. the zone in which stresses are concentrated during detachment), and then discuss how animals might be able to dynamically switch between strong attachment (uniform stress distribution) and easy detachment (stress concentration) during locomotion. Finally, we will discuss possible mechanisms by which animals might increase the efficiency of adhesive pads.

### (a) Stress concentration versus uniform stress distribution

Our results suggest that some climbing animals are able to distribute stresses uniformly within their adhesive contact zones. As the distribution of stress within the contact zone has so far not been measured directly for any natural adhesive system, experimental verification of this important finding is still needed. A uniform distribution of pull-off stresses is achieved when the extension of the peel zone perpendicular to the interfacial crack front is comparable to the size of the adhesive contact. This extension (length) of the peel zone depends on the geometry and stiffness of the adhesive contact.

For example, Hui *et al*. [97] derived an expression for the extension *l* of the peel zone of a fibrillar array with a tape-like flexible backing during a 180° peel7.1
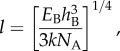
where *h*_B_ and *E*_B_ are the thickness and elastic modulus of the backing, *k* is the spring constant of one fibril and *N*_A_ is the number of fibrils per unit area. Equation (7.1) shows that the length of the peel zone increases both for more compliant fibrils and for stiffer backings. An analogous conclusion can be made using Griffith's criterion [83], here for a flat cylindrical punch geometry7.2
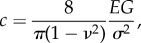
where *c* is the critical crack length, *G* is the effective work of adhesion, *σ* is the strength of the interface, *E* is the elastic modulus and *ν* is Poisson's ratio. The critical crack length increases both for stiffer base materials and for a higher effective work of adhesion. If *c* is comparable to the radius of the contact area, the whole contact will detach simultaneously. Assuming that the flat punch is covered by an adhesive layer or an array of adhesive setae, the effective work of adhesion will increase for softer, viscoelastic adhesive layers (which undergo larger strains and may thus dissipate more energy) or more compliant setae (for a fibrillar array, 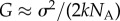). Thus, the critical crack length increases both for more compliant and dissipative adhesives and for stiffer base materials, equivalent to the prediction from equation (7.1).

For large structures, equal load sharing may be achieved by a hierarchical (branched) design with multiple length scales [13,130]. This requires the introduction of an additional level with a different length scale as soon as the spatial extent of the previous (smaller) level approaches the length scale where edge stress concentrations begin to develop. It is possible that similar effects can be achieved in non-fibrillar adhesives, when these include stiffness gradients instead of two dissimilar materials (adhesive and backing). Smooth adhesive pads of insects contain internal branched cuticular fibrils that could convey such a function, and small-scale stiffness gradients near the contact surface have been found both for smooth [27] and hairy pads [28].

**Figure 4.**
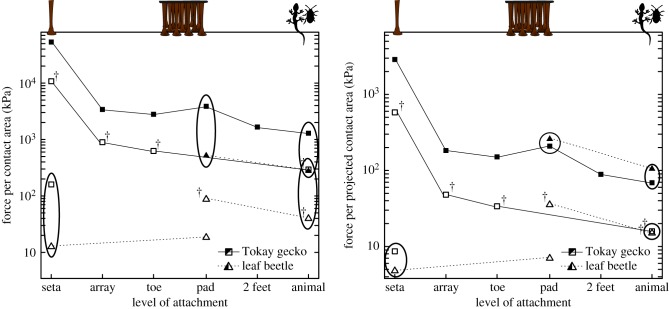
Comparison of attachment stresses at different levels between dock beetles (*Gastrophysa viridula*) and Tokay geckos (*Gekko gecko*). Filled symbols indicate friction, and open symbols depict adhesion per (*a*) ‘real’ or (*b*) projected contact area, respectively. Ellipses highlight comparable measurements at the same level of attachment. The † labels denote adhesion measurements taken in the presence of shear forces. The two species differ by four orders of magnitude in body mass (dock beetles weigh *ca* 10 mg and Tokay geckos up to 100 g). Both frictional and adhesive stresses decrease considerably from the single-seta to the whole-body level for *G. gecko*, but remain approximately constant for *G. viridula*, suggesting that it is difficult for large animals to distribute load uniformly across the adhesive pad contact area, while small animals may be able to achieve a uniform load distribution. While gecko pads generate significantly larger stresses per unit real contact area, the difference disappears when attachment forces are normalized for projected contact area, i.e. the area the animals can effectively use for adhesive footpads. This indicates that gecko pads are not more efficient than beetle pads, despite having much smaller contact sizes (see §§3e, 7c). Details on the sources and calculations underlying this plot are found in the electronic supplementary material.

Nevertheless, uniform loading of the contact zone becomes increasingly difficult for larger animals with larger adhesive organs. There is some indication that geckos, the largest adhesion-based climbing animals, already exceed the upper size limit where equal load sharing is possible, as both frictional and adhesive stresses decrease considerably from the seta to the whole-body level (figure 4 [131]). It has been hypothesized that only a fraction of the setae/spatulae of each toe are in surface contact, explaining this decrease [131,132]. This would imply that only around 6% of all setae were engaged during array-level, and less than 3% during whole-animal level shear measurements. At least for the hairy pads of insects and spiders, contact zone observations showed that virtually all setae can be in surface contact simultaneously on smooth surfaces [101,133], and it is likely that gecko setal arrays are designed to achieve maximum surface contact, too. An alternative explanation for the area-specific decrease in adhesion is that most of the gecko setae/spatulae are in contact, but load is not distributed equally among them (see also [13]). This may be a plausible explanation for the observed decrease in adhesive stress, but it is more difficult to explain the decrease in shear stress, as setae are known to be able to slide smoothly without losing surface contact [129,134], probably mediated by uncorrelated stick–slip of individual spatulae [135,136]. Seta sliding without detachment should rapidly lead to equal load sharing between all setae, inconsistent with the observed loss of shear stress at the array and whole-animal levels. Thus, the existing data suggest that some setae of a gecko array are not in surface contact or that stress concentrations during sliding cause some of the setae to detach, but the details of this process and the mechanisms underlying the observed decrease in stress are still unclear. Direct contact area observations are required to resolve this open question.

In contrast to geckos, dock beetles (*Gastrophysa viridula*) do not appear to show a significant decrease of pull-off stresses from the level of individual setae to the whole body, at least for 90° pull-offs (figure 4 [137]). Although further measurements are required to confirm this conclusion with a fully comparable dataset, this indicates that their contact zones are still small enough to be loaded uniformly, in agreement with equations (7.1) and (7.2). Interestingly, there is evidence that some insects have difficulty detaching their feet when engaging strongly adhesive setae with mushroom-shaped tips [138,139], or after their tarsi were manipulated [140]. While reducing stress concentration likely constitutes the key challenge for larger animals (and for large-area synthetic adhesives), smaller animals may thus face the opposite problem. Even for larger animals, *permanent* area scaling may hamper fast and energy-efficient locomotion, and it is therefore unlikely that the pads produce a uniform stress distribution in all situations, for example during voluntary detachment. Instead, animals may switch between load sharing (strong attachment) and stress concentration (easy detachment), most likely one of the key mechanisms allowing them to combine attachment with locomotion.

### (b) Switching between stress concentration and uniform stress distribution

**Figure 5.**
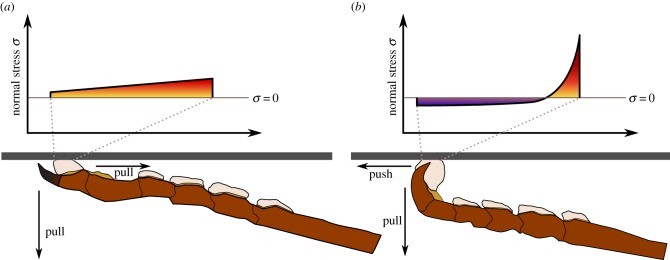
Shear forces may be used to control the normal stress distribution in the contact zone of adhesive pads. When legs are pulled towards the body, normal stresses may be relatively uniform, resulting in strong attachment. If pads are pushed away from the body, the chain-like tarsus may buckle, causing strong stress concentrations at the proximal edge of the pad, facilitating easy detachment. A more detailed discussion of the effect of pushing and pulling on the stress distribution is given in §7b. Note that the figure is not drawn to scale, and stress distributions are shown only schematically.

**Figure 6.**
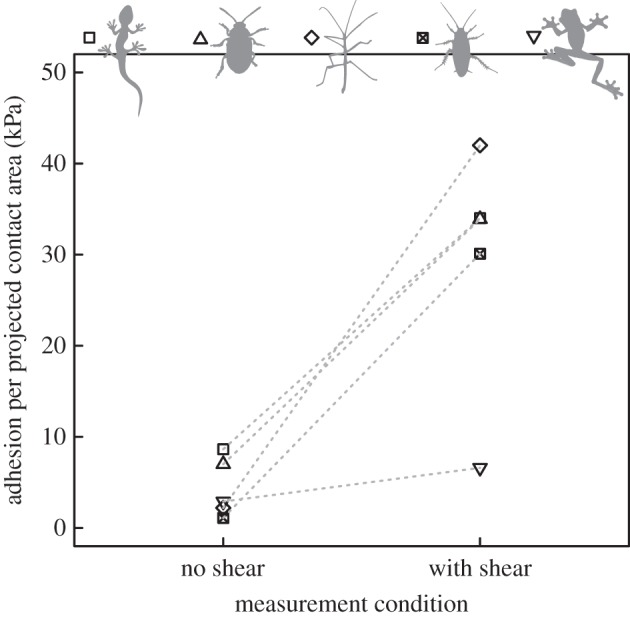
Adhesion per unit projected contact area measured with and without shear force for a Tokay gecko with dry and hairy adhesive pads (*Gekko gecko*, from [129]), a dock beetle with hairy and wet adhesive pads (*Gastrophysa viridula*, from [107,137]), an Indian stick insect (*Carausius morosus*, from [108]), a cockroach (*Nauphoeta cinerea*, from [144] and Y. Zhou 2014, unpublished data), and a tree frog (*Litoria caerulea*, from [145] and N. Crawford 2014, unpublished data), the latter three all with smooth and wet adhesive pads. In all species, adhesive stress increases considerably when shear forces are applied to the pads prior to detachment. Note that the shear-induced adhesive stress shown here might increase even more for higher shear forces (see §7b) and that the magnitude of shear forces differed between the experiments, so that a comparison between the species has to be interpreted with care.

**Figure 7.**
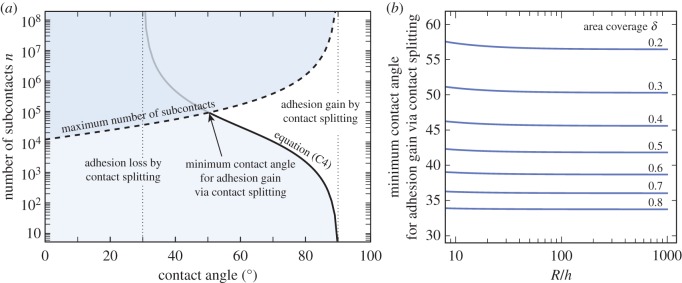
Effect on adhesion if a single meniscus between two flat rigid plates is split into *n* smaller contacts with a size-invariant fluid film thickness. (*a*) Contact splitting will always reduce the adhesive force for contact angles *ϕ* < 30°, and always increase it for 90° < *ϕ* < 150°. For 30° < *ϕ* < 90°, the effect of contact splitting will depend on the number of sub-contacts *n*, the area coverage *δ*, the ratio *R*/*h* between radius and fluid film thickness, as well as on the contact angle *ϕ* according to equation (C4) (limit indicated by the solid line). If the fluid film thickness is size-invariant, *n* has an upper limit (indicated by the dashed line), which further restricts any adhesion gain via contact splitting to even larger angles, as indicated by the intersection of the dashed and the solid line. The plot shows the number of sub-contacts required to increase adhesion via contact splitting for *δ* = 0.3 and *R*/*h* = 100. (*b*) Minimum angle for which an adhesion gain via contact splitting is possible (i.e. the angle where the dashed and the solid lines in (*a*) intersect), plotted as a function of *R*/*h* and *δ*. (Online version in colour.)

Some insects can generate whole-body attachment forces equivalent to more than 100–300 times their own weight [109,128,141], and it is intriguing that they are nevertheless able to detach their feet rapidly, and climb efficiently. As uniform stress distribution (area scaling) results in strong adhesion, whereas stress concentrations (length scaling) facilitate easy detachment, this observation suggests that climbing animals are able to switch between both states. This ability may be an essential prerequisite for combining reliable surface attachment with rapid locomotion, but the detailed underlying mechanisms are still unclear. The switching is likely controlled at different levels, ranging from single setae/spatulae to the whole animal, and there is strong evidence that shear (pushing and pulling) forces are essential (figure 5). Stress concentrations can be *enforced* by pushing the pads away from the body causing the tarsal joints to buckle [57,107] or by specialized detachment movements such as ‘hyperextension’ of individual toes [142]. When legs are *pulled* towards the body, in contrast, the contact is stabilized and the strength of the interface increases significantly [57,73,107,108,143] (figure 6). Indeed, pulling forces may help to *reduce* (tensile) stress concentrations—and thus influence the scaling of adhesive forces—for several reasons:

First, it has been suggested that pulling forces can increase the length of the peel (cohesive) zone both at a microscopic level for spatula adhesion and at a macroscopic level for tape peeling, so that stresses are distributed over larger fractions of the total available contact area which in turn increases the adhesive force [70,146,147]. Second, if pad adhesion follows peeling theory, strong attachment forces would require the maintenance of low peel angles, and thus large shear forces. Pads pulled horizontally will eventually slide, and forces will be limited by the pad's maximum shear stress [73]. Assuming a body size-independent shear stress, maximum possible adhesion for friction-limited peeling scales with 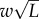, and thus with *m*^0.5^—intermediate between length- and area-scaling—while forces would scale with length for a 90° pull-off (see appendix D). The different contributions of *w* and *L* to peak attachment forces at small pull-off angles may explain why adhesive contact areas are wider than long in many insects (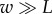, see e.g. [108]). Interestingly, no such trend seems to be present for the shape of individual adhesive hairs. Third, the effect of shear forces may be explained by the moments and ensuing stress concentrations introduced into the contact zone when oblique seta tips with stiff stalks come into contact by bending. While a perpendicular pull-off will concentrate tensile stresses at the proximal edge of the pad contact zone, a simultaneous horizontal pull will reduce or remove these stresses, thereby increasing adhesion [69,148]. Fourth, the anisotropy of biological adhesive materials can give rise to a longer peel zone when pulls are aligned with the stiffest axis, leading to direction-dependent adhesion [130]. Fifth, shear forces could induce a change in the adhesive material's overall stiffness, or the direction of its stiffest axis (see equations (7.1) and (7.2)). A smooth transition from length to area scaling has been demonstrated in detailed finite-element computations of detaching gecko spatulae with increasing bending stiffnesses [72]. The angle of the internal fibrils of smooth adhesive pads is reduced when pads are pulled towards the body [149], thereby potentially changing the pad's stiffness and expanding the peel zone. However, all these hypotheses remain to be tested experimentally.

In general, the coupling between adhesion and friction (termed ‘frictional adhesion’ or ‘shear-sensitive adhesion’ in previous work) observed in ‘wet’, ‘dry’, ‘hairy’ and ‘smooth’ biological dynamic adhesives remains insufficiently explained, but it is of central importance for rapidly controllable attachment in climbing animals (figure 6). Current evidence suggests that adhesion increases approximately linearly with friction over nearly seven orders of magnitude [108,129,143] and it is unclear whether this relationship holds up to the maximal achievable friction, or breaks down earlier. For example, adhesive stress measured in the absence of shear forces in wet adhesive pads of Indian stick insects is only 2 kPa [108], but peak adhesive stresses can exceed 200 kPa (D. Labonte 2014, unpublished data). In geckos, single setae show an adhesive stress of only 8 kPa when detached without being simultaneously dragged across the surface, but peak adhesive stresses measured while the setae were sheared are close to 0.6 MPa ([129]; see the electronic supplementary material). In both cases, the increase of adhesive strength with shear forces is approximately two orders of magnitude, which has two important implications: first, theoretical models for controllable attachment in biological adhesives have to explain the strong change of adhesion with shear forces. Second, comparisons between different attachment systems or experimental conditions have to be treated with caution if the magnitude of shear stresses acting during detachment was not comparable. Clarifying the mechanisms leading to the approximately linear relationship between adhesion and friction is also important for understanding the scaling of biological adhesives, as it implies area scaling of adhesion, independent of contact size.

### (c) Increasing pad efficiency: strength and toughness

Our data indicate that within some animal taxa (with smooth pads, no data are yet available for hairy pads), adhesion grows faster than pad area, i.e. the pads' efficiency increases with body size. This increase in adhesive strength is important for climbing animals, and may also be relevant for the design of synthetic adhesives. However, the underlying mechanisms are still unclear.

For fibrillar adhesives, ‘force scaling’ could provide an explanation for changes in adhesive strength [68,86], but at least two arguments speak against an important role of this effect in natural adhesive systems. First, there is no experimental evidence that the stresses of biological adhesives pads increase with decreasing contact size (e.g. [126]). Dock beetle setae are comparable in size and adhesive force to single gecko setae, yet a gecko seta consists of approximately 250 spatulae with more than 200 times smaller tips (figure 4*b* and electronic supplementary material, table S2). This finding indicates that the force per real contact area may increase for smaller contacts, but this gain is fully balanced by the less efficient use of the available foot surface area. This less efficient area use may be based on the smaller area coverage, and/or on incomplete surface contact of the spatulae. Second, contact splitting does not explain the variation of hair density observed between animals of different body sizes and taxa. By analysing data from 81 animal species with hairy attachment pads, Peattie & Full [111] showed that when evolutionary relationships were accounted for, spatula density did not change significantly with body mass within groups of related taxa, and variation was mainly explained by evolutionary history (see also [11,117]).

Among the different adhesion models surveyed in this review, only viscous (Stefan) adhesion predicts an increase of strength with size, but the assumptions of rigid substrates and size-invariant fluid film thickness may be unrealistic for natural adhesives. Other possible explanations for an increase of adhesive strength with size would require size-dependent changes of relevant pad properties. For example, the strength of ‘wet’ adhesive systems could be enhanced by reducing the amount of fluid secreted or increasing its viscosity (cf. electronic supplementary material). Moreover, adhesion could be increased in larger animals via the same factors that increase the length of the pad's peel zone, i.e. by increasing the effective work of adhesion and backing stiffness (cf. equations (7.2) and (3.1)). To our knowledge, however, hardly any evidence exists to date to support or reject these hypotheses.

In hylid tree frogs, epithelial cell area increased with body size, and this was found to correlate with an increased pad efficiency [121]. This finding could be explained by an ‘inverse contact splitting’ effect; assuming that adhesive strength is dominated by viscous (Stefan) adhesion of epithelial cells (with facilitated fluid flow through the channels between them), increasing epithelial cell area would enhance adhesive efficiency.

Other possible ways to increase the pads' effective work of adhesion include the introduction of surface patterns or elastic inhomogeneities close to the interface that will act as crack arresters [150–158]. In fibrillar adhesive systems, the effective work of adhesion can be increased by making setae longer or thinner and thereby more compliant. A possible example for an intraspecific increase in seta compliance with size is given by the gecko *Chondrodactylus bibronii*, where seta length was found to increase with body size while seta density and diameter remained constant [117].

The effectiveness of the aforementioned mechanisms depends on whether pads are in the regime of uniform load distribution. Mechanisms that increase the toughness of the interface will not increase adhesion if the loading is uniform, as in this case adhesion is solely determined by the strength of the interface. It remains unclear whether biological adhesives have to be particularly strong, tough, or both.

## 8. Conclusion

Large body size is expected to be in conflict with surface attachment by claws or adhesive pads, but the wide size range of climbing animals suggests that adaptations compensating for these problems have evolved. Possible adaptations of claws for larger body sizes are still unclear and require further study. For the scaling of adhesion, our results indicate that the clinging performance of some climbing animals is approximately size-independent, contrary to predictions from isometry. The available data suggest that not only are many climbing animals able to minimize stress concentrations within the contact zone of their adhesive pads, but also in some cases adhesive efficiency (force per unit contact area) increases with body size. As the currently available intraspecific scaling data suggest isometry or even negative allometry of adhesive pad area, this increase in adhesive strength is required to compensate for the weight-specific decrease of adhesion and is thus of high biological relevance. However, the underlying mechanisms are still completely unclear and have to be addressed in future research.

It is likely that a key requirement for rapidly controllable adhesives is the ability to switch between area and length scaling, and shear forces are essential in this process. Animals can minimize pulling forces when detaching feet individually, but forced pull-offs will automatically result in an inward pull due to the sprawled leg posture of climbing animals. Thus, the performance of adhesive pads does not solely depend on their isolated properties, but also on the way they are used for locomotion at the whole-animal level.

Natural adhesive systems promise to provide inspiration for novel synthetic adhesives, but many fundamental questions about their function are still unresolved. For example, it is largely unclear how the size and density of setae/spatulae affect the adhesive performance in climbing animals. It is also unknown how stresses are distributed in the contact zone and what proportion of adhesive hairs are in surface contact during locomotion. Moreover, a mechanistic understanding of the widespread coupling between friction and adhesion is still lacking. For many other parameters of adhesive pads, it is unknown how they depend on size, such as the dimensions of the internal fibrils and the outer layer in smooth pads, the material properties of adhesive pads, as well as the volume and properties of their adhesive secretions. Information on the variation of these parameters with body size, and on their effects on adhesive performance will help to clarify how large animals can maintain strong attachment and improve our understanding of biological adhesive mechanisms.

## Supplementary Material

Supplementary information

## Appendix A. Scaling of claw stress

The maximum force the claw of a climbing animal has to support may be proportional to the mass of the animal *m*. When a conical (solid or hollow) claw tip catches on an asperity, the stresses generally decrease from the tip towards the claw base (as the moment of inertia grows faster with distance from the claw tip than the bending torque). At the contact point of the tip, claw material and substrate are sheared and compressed, and both can fail [30]. The area on which this shear force is acting is proportional to the square of the claw tip radius *R*_CT_ [30], which for isometric animals scales with *m*^1/3^. This indicates that the risk of claw tip failure increases with body size for isometric animals, although the claw tips are larger, as . Maintaining a constant stress *σ* at the claw tips requires a positive allometric growth of the claw tip radius

A 1

which indicates that *R*_CT_ ∝ *m*^0.5^ for the tip stress to be constant.

## Appendix B. The importance of fluid film thickness for the scaling of ‘wet’ adhesives

The scaling of adhesion for ‘wet’ contacts depends not only on the geometry and stiffness of the contact, but also on the relationship between contact size and fluid film thickness. In order to maximize adhesion, insects should secrete as little fluid as possible on smooth surfaces, but on rough surfaces the secretion film should be thick enough to fill the gaps between the pad and the surface [106]. The dependence of fluid film thickness on contact size may thus be estimated by assuming that the amount of fluid is sufficient to compensate the surface roughness amplitude of a self-affine rough surface. The statistical properties of self-affine surfaces do not change for the transformation

B 1

where *ζ* is a factor representing the ‘magnification’, (*x*, *y*) is a two-dimensional position vector in the surface plane and *H* is the Hurst exponent (0 ≤ *H* ≤ 1). Equation (B 1) implies that if *R* is the radius of a contact element, and fluid film thickness *h* corresponds approximately to the roughness amplitude over the area of the contact element, *h* should scale as *R^H^*. Thus, for different values of *H*, the dependence ranges from size-invariant fluid film thickness (*H* = 0) to a linear proportionality between *h* and *R* (*H* = 1).

For the two limiting values of *H*, different mechanisms may limit adhesive strength. If the fluid film thickness is size-invariant (*H* = 0), the meniscus has a minimum diameter of 2*R* > *h*(1 − sin*ϕ*)/cos*ϕ*. For a linear relationship between fluid film thickness and contact size (*H* = 1), the maximum adhesive strength is set by the tensile strength of the liquid. For strongly negative pressures—e.g. during rapid pull-offs—the liquid may fail in tension by cavitation. Estimates for the critical pressure range from a few to several hundred MPa [92,159,160], so that the maximum strength for a ‘wet’ contact may be comparable to that for van der Waals forces.

To date, nothing is known about the effect of contact size on secretion volume in biological adhesive systems, and further research is required to clarify which scaling assumption is most realistic.

## Appendix C. Contact splitting for ‘wet’ adhesives

For two flat, rigid plates, separated by a disc-shaped, circular film of liquid, the (static) adhesive force is the sum of two components: the curvature of the meniscus induces a Laplace pressure difference and the resulting force scales with area. The second force component arises from the surface tension of the liquid acting along the perimeter, and therefore scales with length. The total force adhesive *F* is

C 1

where *R* is the radius of the disc-shaped liquid film, *γ* is the surface tension of the liquid and *ϕ* is the contact angle with the substrates (for simplicity, we assume that the contact angles with the two plates are the same). For two parallel surfaces, Δ*p* = *γ* (2cos*ϕ*/*h* − 1/*R*) (Δ*p* > 0; pressure within the fluid meniscus is lower than outside), where *h* is the thickness of the fluid film. Equation (C 1) becomes

C 2

If the single contact is replaced by *n* small contacts of radius *r*, with area coverage *δ* = *n*(*r*/*R*)^2^, the new force *F*_cs_ is

C 3

Equation (C 3) is valid if the fluid film thickness is independent of the radius of the individual contacts, so that contact splitting would result in a decrease of the fluid volume by a factor of ≈*δ*. Alternatively, fluid volume could be conserved, in which case *h* would increase to ≈*h*/*δ*. In both cases, contact splitting will always decrease (static) adhesion if *ϕ* < 30°, as then the length-dependent term on the right-hand side is negative. For 90° < *ϕ* < 150°, contact splitting will always increase adhesion, as now the length scaling term is positive and the area scaling term is negative. For 30° < *ϕ* < 90°, the two terms compete—contact splitting might result either in an increase or a decrease in adhesion, depending on the number of hairs *n*, the area coverage *δ*, the ratio *R*/*h* between total contact radius and fluid film thickness, as well as on the contact angle *ϕ*. Assuming that the forces of biological adhesives are described by equation (C 3), it is implausible that they benefit from contact splitting, as adhesive secretions of insects, spiders and tree frogs mostly show contact angles of less than 30° [99–101].

Equating equations (C 2) and (C 3), and solving for *n* indicates that the minimum number of hairs required for adhesion gain via contact splitting is

C 4

(for constant *h*) or

C 5

(for constant volume).

The transition between loss and gain of adhesion as a result of contact splitting is shown in figure 7*a* for *δ* = 0.3 and *R*/*h* = 100 (constant *h*).

As noted above, contact splitting with a size-invariant fluid film thickness (*H* = 0) is limited by the minimum diameter of sub-contacts 2*R* > *h*(1 − sin*ϕ*)/cos*ϕ* which implies

C 6

This further limits adhesion gain via contact splitting, as illustrated in figure 7*b*, which shows the contact angle calculated by combining equations (C 4) and (C 6)—the smallest contact angle for which an adhesion gain via contact splitting is possible for a given combination of *R*/*h* and *δ*.

If the relationship between the size of individual contacts and the fluid film thickness is linear (i.e. *H* = 1) with a proportionality constant *k* = *r*/*h* (*k* > 1), equation (C 3) becomes

C 7

Now, both the Laplace pressure and the surface tension term scale with length. Equating equations (C 2) and (C 7) and solving for *n* yields

C 8

Thus, if fluid film thickness is proportional to a linear dimension of the individual contacts, contact splitting will increase the adhesive force by a factor , as long as *n* > 1/*δ* and *k*cos(*ϕ*) + sin(*ϕ*) − 0.5 > 0. The latter condition is always met for *ϕ* < 90° (as *k* ≥ 1), but will depend on *k* for larger contact angles.

The above considerations are limited to the effect of splitting *one* large contact into multiple smaller ones, where the area loss associated with contact splitting can be a dominant factor. The situation is different, however, if one considers splitting  contacts into *n*_2_ smaller contacts, as here area coverage can be preserved. In such a situation, decreasing contact size will increase adhesion if the term scaling with length is positive (see equation (C2)). In terms of the design of ‘wet’ adhesive pads, this indicates that fibrillar structures may benefit from smaller contact sizes, but they do not necessarily outperform one continuous contact in terms of adhesive strength.

## Appendix D. Scaling prediction for friction-limited peeling

The adhesion of footpads of geckos, tree frogs and insects was found to increase with the lateral (pulling) forces acting during detachment [73,108,143]. The detailed mechanisms underlying this increase are still unclear, but a frequent approach is to model adhesive pads or setae as stripes of thin tapes (e.g. [70–73]). The force required to detach a thin, inextensible tape depends on the tape width *w*, the effective work of adhesion *G* and the peel-angle *α* [62,63,161].

D 1

where it is assumed that inertial forces and bending energy are negligible and that peeling is in a steady-state mode. Equation (D 1) predicts that *F* scales with length and thus as *m*^1/3^. Large detachment forces require small peel angles, as for *a* → 0, *F* → ∞. Consistently, climbing animals pull their leg inwards when large adhesion is required [52,73,108,141], increasing the shear forces acting on their pads and decreasing the peel angle. Equation (D 1) can be re-written as a function of the friction force *F*_F_, using trigonometric relationships

D 2

Assuming that pad adhesion can be described by this simple model, adhesion is limited by the maximal sustainable friction force. Friction will scale with the pad's contact area *A* = *wL*, where *L* is the proximal–distal length, and *w* is the transverse width of the contact zone. We find

D 3

where *τ* is the maximum sustainable shear stress of the pads, which will depend on the sliding velocity. Estimated values for insects (*G* ≈ 100 mN m^−1^, *τ* ≈ 500 kPa; *L* ≈ 100 µm) suggest that  so that the force should scale with , corresponding to *m*^0.5^, i.e. intermediate between length and area scaling.
