# Supplementary material for: Scaling and biomechanics of surface attachment in climbing animals
Source: Philos Trans R Soc Lond B Biol Sci. 2015 Feb 5;370(1661):20140027. doi: 10.1098/rstb.2014.0027 (PMC4275900; doi:10.1098/rstb.2014.0027)
Supplement: Supplementary information [file rstb20140027supp1.pdf]

Scaling and biomechanics of surface attachment in climbing animals

Supplementary information

David Labonte & Walter Federle

Department of Zoology, University of Cambridge

Wet adhesion and viscosity

The presence of a liquid cannot only influence adhesion via surface tension, but also via viscosity. The force required to separate two rigid, parallel disks of radius R immersed in a Newtonian liquid is given by Stefan (1875) [1]

$$F_{disc} = \frac{3}{2}\pi\eta\frac{R^4}{h^3}\frac{dh}{dt} \tag{1}$$

where η is the liquid's viscosity, h is the thickness of the liquid-filled gap between the disks and dh/dt is the pull-off velocity. Note that F_{disc} is zero in the static case.

If h is independent of pad (disk) size ($H = 0$), Eq. 1 predicts forces to scale with R^4, i. e. with the square of the contact area, or with $m^{4/3}$. If R/h is constant ($H = 1$), however, forces will scale with length and thus $m^{1/3}$.

A similar model for the viscous adhesion of a sphere immersed in a Newtonian fluid is given in Francis and Horn [2]

$$F_{sphere} = 6\pi\eta\frac{R^2}{h}\frac{dh}{dt} \tag{2}$$

where h is defined as the minimum fluid film thickness underneath the centre of the sphere. If h is independent of R ($H = 0$), Eq. 2 predicts area scaling. If R/h is constant ($H = 1$), Eq. 2 predicts length scaling.

Measurement of pad area and adhesive force in different-sized leaf-cutting ants (*Atta colombica*)

Workers of leaf-cutting ants (*Atta colombica*, n=115, 0.37-43.40 mg body mass) were used to study the allometry of adhesion and pad area. The contact area of the ants' smooth adhesive pad (arolium) was measured separately for front, middle and hind legs in 31 workers. The arolium is a highly flexible structure and has to be unfolded to achieve full contact area [3].

Ants were held by their thorax with insect handling tweezers and pulled across a glass slide mounted on a Zeiss Axiovert 135 (inverted) microscope. For legs where shear forces corresponded to a pull towards the body, the arolium fully unfolded. Contact areas were viewed at 10x magnification with reflected light illumination, and filmed using a Redlake (San Diego, USA) PCI 1000 B/W high-speed video camera. The contact area of unfolded arolia was measured from selected frames using ImageJ [4].

Adhesion forces of 84 leaf-cutting ants were measured using a centrifuge technique [5, 6]. Ants were placed onto the surface of a smooth PMMA cylinder (radius 40 mm) mounted in the rotor of a centrifuge. The centrifuge was gradually accelerated until the insect detached. A standard interlaced CCD video camera (Panasonic F15) with 50 Hz half-image frequency filmed the rotor from above (distance 0.9 m). The centrifuge was illuminated with a 100 Hz strobe light so that two ants were visible in each video half-frame. We digitized the ant's positions just before detachment, allowing us to calculate centrifugal acceleration from the ant's radius on the centrifuge and the angular speed of rotation. Each ant was weighed in a microgram balance, and detachment force was calculated as the product of body mass and centrifugal acceleration. For each ant worker, we used the maximum force from three consecutive measurements (allowing at least 15 min recovery time between repeats).

The scaling of pad area and whole body adhesion with body mass was analysed using standardized major axis (SMA) regression on log-transformed values (see Tab. 1 for results), which is more appropriate than ordinary least squares (OLS) in allometric studies where the variables of interest often have no causal relationship and are both measured with some error [7]. For some animals – such as ants in particular – body mass can vary strongly as a result of the filling state of the crop. Statistical analyses were conducted using the *smatr* package and Rv3.1.0 [8, 9]. The *smatr* package also provides methods for statistically comparing SMA slopes among groups (front, middle and hind legs), as well as a statistical test for whether the SMA slope differs significantly from an expected value [7].

Table 1 | Scaling of contact area and adhesive performance in leaf-cutting ants. Results of standardised major axis (SMA) regressions of log-transformed adhesion and pad area against log-transformed body weight.

	n	Scaling coefficient (95% CI)	R^2	Statistical test for area scaling
Whole body adhesion	84	0.98 (0.92-1.04)	0.92	r_{82}=0.80, p<0.001
Contact area		0.59 (0.54-0.60)	0.93	r_{89}=-0.51, p<0.001
front legs	31	0.61 (0.55-0.67)	0.94	r_{29}=-0.36, p<0.05
middle legs	29)	0.55 (0.50-0.61)	0.94	r_{27}=-0.62, p<0.001
hind legs	31	0.55 (0.49-0.62)	0.90	r_{29}=-0.54, p<0.01

Table 2 | Friction, adhesion and contact area measured at different levels for a leaf beetle and a gecko species (see Fig. 3 in the main manuscript). For geckos, projected contact area was converted into real contact area assuming that all spatulae were in contact during the measurements, and that the spatula density was $3.6 \cdot 10^6$ mm^{-2} [10]. The contact area per spatula was estimated by assuming a triangular shape of 200 nm width, and 150 nm height, yielding an area of $0.015 \mu m^2$. The number of spatulae per seta was calculated as the quotient of spatula and seta density (14400 mm^{-2} [11]), yielding 250 spatulae per seta. Note that the small difference in contact area between the toe and pad measurement in *Gekko gecko* is due to the use of a small gecko in [12]. For leaf beetles, we assumed that the whole animal contact area is equal to the summed area of all distal pads as these likely detach last, and therefore set the limit to the adhesive strength [13]. Real contact area was converted into projected contact area using an area coverage of 38% [14]. The small decrease in shear stress from the single pad to the whole animal level in dock beetles is likely explained by the fact that the beetles cannot align all legs with the centrifugal force in order to ensure maximum shear resistance. The † labels denote adhesion measurements that were taken in the presence of shear forces, so that comparisons to 90° pull-offs have to be drawn with care.

Species	Friction [mN]	Adhesion [mN]	Proj. contact area [mm^2]	Real contact area [µm^2]	Source	Level
Gastrophysa viridula	-	0.00058	0.0001218	46.3	[14]	Seta
(body weight $\approx$ 10 mg)	11.1	0.30/1.78†	0.0429	16300	[15, 14]	Pad
	27.04	3.88†	0.26	97800	[16, 14]	Animal
Gekko gecko	0.2	0.0006/0.04†	0.00006946	3.75	[17, 18]	Seta
(body weight $\approx$ 60 g)	171	45 †	0.93	50220	[19]	Array
	2874	644 †	19	1026000	[18, 20]	Toe
	4600	-	22	1188000	[12]	Pad
	20400	-	227	12258000	[21]	Two pads
	31220	7090†	447	24138000	[22, 23]	Animal

ADHESION MEASURED WITH AND WITHOUT SHEAR FORCES

Table 3 | Adhesive stress (adhesion per projected contact area) measured with and without shear (pulling) forces acting during detachment (used for Fig. 4 in the main manuscript). Independent of pad morphology ('hairy' or 'smooth') and pad type ('wet' or 'dry'), adhesive stress is considerably larger when measured in the presence of pulling forces. Note that direct comparisons between the species have to be drawn with care, as the experimental conditions differed. The adhesive stress measured in the presence of pulling forces reported here is likely not the upper limit, but set by the experimental protocol. For example, adhesive stresses in stick insects in [24] were measured after a shear force of 8 mN was applied to the pads but peak stresses can reach more than 200 kPa when larger pulling forces are applied prior to detachment (D Labonte, unpublished data).

Species	Adhesive stress without shear [kPa]	Adhesive stress with shear [kPa]	Source
Gastrophysa viridula	7	35.5	[15, 14]
Gekko gecko	8.6	33.9	[17, 20]
Carausius morosus	2.2	42.2	[24]
Litoria caerulea	2.9	6.6	[25], N Crawford
Nauphoeta cinerea	1.1	30.1	[26], Y Zhou

REFERENCES

[1] Stefan J. 1875 Versuche über die scheinbare Adhäsion. *Annalen der Physik* **230**, 316–318.

[2] Francis B, Horn RG. 2001 Apparatus-specific analysis of fluid adhesion measurements. *J Appl Phys* **89**, 4167–4174.

[3] Federle W, Brainerd EL, McMahon TA, Hölldobler B. 2001 Biomechanics of the movable pretarsal adhesive organ in ants and bees. *PNAS* **98**, 6215 – 6220.

[4] Schneider C, Rasband W, Eliceiri K. 2012 Nih image to imagej: 25 years of image analysis. *Nat Methods* **9**, 671–675.

[5] Federle W, Rohrseitz K, Hölldobler B. 2000 Attachment forces of ants measured with a centrifuge: better 'wax-runners' have a poorer attachment to a smooth surface. *J Exp Biol* **203**, 505–512.

[6] Federle W, Baumgartner W, Hölldobler B. 2004 Biomechanics of ant adhesive pads: frictional forces are rate- and temperature dependent. *J Exp Biol* **206**, 67–74.

[7] Warton DI, Wright IJ, Falster DS, Westoby M. 2006 Bivariate line-fitting methods for allometry. *Biological Reviews* **81**, 259–291.

[8] Warton DI, Duursma RA, Falster DS, Taskinen S. 2012 smatr - an r package for estimation and inference about allometric lines. *Methods in Ecology and Evolution* **3**, 257–259.

[9] R Development Core Team. 2013 R: A Language and Environment for Statistical Computing. R Foundation for Statistical Computing, Vienna, Austria. URL `http://www.R-project.org`. ISBN 3-900051-07-0.

[10] Peattie AM, Full RJ. 2007 Phylogenetic analysis of the scaling of wet and dry biological fibrillar adhesives. *PNAS* **104**, 18595–18600.

[11] Schleich HH, Kastle W. 1986 Ultrastrukturen an Gecko-Zehen (Reptilia: Sauria: Gekkonidae). *Amphibia-Reptilia* **7**, 141–166.

[12] Autumn K, Peattie AM. 2002 Mechanisms of adhesion in geckos. *Integr Comp Biol* **42**, 1081–1090.

[13] Clemente CJ, Federle W. 2008 Pushing versus pulling: division of labour between tarsal attachement pads in cockroaches. *Proc R Soc B* **275**, 1329–1336.

[14] Bullock J, Federle W. 2011 Beetle adhesive hairs differ in stiffness and stickiness: *in vivo* adhesion measurements on individual setae. *Naturwissenschaften* **98**, 381–387.

[15] Bullock JMR, Drechsler P, Federle W. 2008 Comparison of smooth and hairy attachment pads in insects: friction, adhesion and mechanisms for direction-dependence. *J Exp Biol* **211**, 3333–3343.

[16] Gorb E, Gorb S. 2009 Effects of surface topography and chemistry of rumex obtusifolius leaves on the attachment of the beetle gastrophysa viridula. *Entomol Exp Appl* **130**, 222–228.

[17] Autumn K, Liang Y, Hsieh S, Zesch W, Chan W, et al.. 2000 Adhesive force of a single gecko foot-hair. *Nature* **405**, 681–685.

[18] Autumn K. 2006 Properties, principles, and parameters of the gecko adhesive system. In: Biological adhesives (ed. Smith A, Callow J), pp. 225–256. Springer, Berlin.

[19] Gravish N, Wilkinson M, Autumn K. 2008 Frictional and elastic energy in gecko adhesive detachment. *J R Soc Interface* **5**, 339–348.

[20] Guo C, Sun J, Ge Y, Wang W, Wang D, et al.. 2012 Biomechanism of adhesion in gecko setae. *Sci China Life Sci* **55**, 181–187.

[21] Irschick DJ, Austin CC, Petren K, Fisher RN, Losos JB, et al.. 1996 A comparative analysis of clinging ability among pad-bearing lizards. *Biol J Linnean Soc* **59**, 21–35.

[22] Niewiarowski PH, Lopez S, Ge L, Hagan E, Dhinojwala A. 2008 Sticky gecko feet: the role of temperature and humidity. *PLoS One* **3**, e2192.

[23] Pugno N, Lepore E, Toscano S, Pugno F. 2011 Normal adhesive force-displacement curves of living geckos. *The Journal of Adhesion* **87**, 1059–1072.

[24] Labonte D, Federle W. 2013 Functionally different pads on the same foot allow control of attachment: stick insects have load-sensitive "heel" pads for friction and shear-sensitive "toe" pads for adhesion. *PLoS One* **8**, e81943.

[25] Crawford N, Endlein T, Barnes WJP. 2012 Self-cleaning in tree frog toe pads; a mechanism for recovering from contamination without the need for grooming. *J Exp Biol* **215**, 3965–3972.

[26] Dirks JH. 2009 Mechanisms of fluid-based adhesion in insects. Ph.D. thesis, University of Cambridge.
